# Influence of Waterproof Films on the Atomization Behavior of Surface Acoustic Waves

**DOI:** 10.3390/mi10110794

**Published:** 2019-11-19

**Authors:** Qing-Yun Huang, Hong Hu, Jun-Long Han, Yu-Lin Lei, Xiao-Qing Yang

**Affiliations:** School of Mechanical Engineering and Automation, Harbin Institute of Technology, Shenzhen, Guangdong 518055, China

**Keywords:** surface acoustic wave (SAW), film coating, atomization behavior

## Abstract

One of the reasons why commercial application of surface acoustic wave (SAW) atomization is not possible is due to the condensation of aerosol droplets generated during atomization, which drip on the interdigitated transducer (IDT), thereby causing electrodes to short-circuit. In order to solve this problem, a SU-8-2002 film coating on an IDT is proposed in this paper. The waterproof performance of the film coating was tested on a surface acoustic wave (SAW) device several times. The experimental results reveal that the film coating was robust. The experiment also investigated the effects of the SU-8-2002 film on atomization behavior and heating.

## 1. Introduction

The generation of micron- and submicron-sized sprays is important for many industrial processes. Dense, monodisperse aerosols with a controllable size are widely employed in many applications, such as fuel injection, agriculture sprays, mass spectrometry, ink-jet printing, and pulmonary drug delivery. The essence of atomization is that external forces overcome the capillary force within the fluid and decompose into aerosol droplets. Common atomization methods include pressurized nozzles, electrospraying, ink-jets, and ultrasonic atomization. Among these methods, ultrasonic atomization has been widely investigated and utilized.

Since the first description of ultrasonic atomization by Wood and Loomis et al. [1], a series of studies have focused on ultrasonic atomization mechanisms and characteristics. Lang et al. [2] used alternating signals to drive piezoelectric transducers, thereby generating ultrasonic and measured the droplet sizes from the breakup of the capillary wave on the liquid surface; they concluded that the droplet size was directly proportional to the wavelength of the capillary waves. Lang et al. also proposed a formula describing the relationship between the droplet size and excitation frequency, which showed a tendency for the droplet size to decrease with increasing excitation frequency [2]. However, due to material limitations, excitation frequency was limited to 3 MHz. In biological applications, this low frequency would damage molecules. Although the ultrasonic atomizer is small compared to other atomizers, it is difficult to miniaturize to the point where it can be hand-held.

Kurosawa et al. [3,4] proposed a new type of ultrasonic atomizer using a surface acoustic wave (SAW) device. A SAW is a nanometer-order amplitude acoustic wave. SAW technology has been widely used in filters, biosensors, wireless transmission, and radio frequency (RF) duplexers and has potential applications in the field of microfluidics. Its role is to propagate along the surface of a substrate and decay within one or two wavelengths into the substrate depth, with little loss in the direction of the surface propagation. The most common SAW use in microfluidics is the Rayleigh wave, which attenuates a leaky surface acoustic wave (LSAW) when it comes into contact with droplets, due to a mismatch of sound velocity in the fluid (about 1485 m/s for water) and piezoelectric substrate (3965 m/s for 128° Y–X lithium niobate). The LSAW attenuates along the propagation path, and that diffracts a longitudinal wave enters the droplet at the Rayleigh angle, *θ_R_*. It attenuates in a viscous fluid medium, then the longitudinal wave propagates within the droplet, generating a pressure gradient that causes acoustic flow within the droplet. As the power increases, the droplet shows a series of dynamics, such as vibration, pumping, jetting, and atomization. When the input power reaches a threshold value, a capillary wave is generated on the surface of the droplet and starts to break up, subsequently forming atomization. In order to achieve high performance using SAW devices, the choices of Piezo substrate materials are significant. The most common materials are lithium tantalate (LiTaO_3_), lithium niobate (LiNbO_3_), zinc oxide (ZnO), and aluminum nitride (AlN). LiNbO_3_ crystals characteristically have a large electromechanical coupling coefficient and a small propagation loss, so they are suitable for fabricating low-loss SAW devices with broadband. So far, this material has been widely used in SAW devices. LiTaO_3_ crystals have a large electromechanical coupling coefficient similar to LiNbO_3_, but the temperature stability of LiTaO_3_ crystals is better than lithium niobate crystals. However, LiTaO_3_ crystals have a high melting point, complex production equipment and technology, and high production costs. Since the 1960s, the development of piezoelectric and ferroelectric thin films has made it possible for piezoelectric thin films to be used in SAW devices. At present, more research is being carried out regarding zinc oxide and aluminum nitride deposition onto diamond films. Among them, the ZnO SAW device is relatively mature. Compared with the traditional ultrasonic atomizer, it has a higher excitation frequency and consequently produces smaller aerosol droplets. The linear mean diameter in a SAW atomizer was found to be 19 μm under an excitation frequency of 10 MHz [3,4]. Wang and Hu measured the atomized droplet size distribution generated by a SAW device in the order of 20 MHz. Analysis by a laser diffraction system revealed that the diameter distribution of droplets centered on three main areas, namely, 1 μm, 10 μm, and 100 μm [5]. Ju and Yamagata investigated a high-frequency SAW atomizer with resonance frequencies ranging from 50 to 95 MHz and found that the mean diameters of atomized droplets were 5.7 μm (50 MHz), 4.4 μm (75 MHz), and 2.7 μm (95 MHz) [6]. Unlike an ultrasonic atomizer, where the energy is transferred in the form of a large block, the energy transfer of the SAW was localized on the substrate. The power required was therefore much less than what was required for ultrasonic atomization. It is possible to achieve atomization at 2–3 W of power, which allows the device to be hand-held; therefore, this was considered as an ideal device for applications such as pulmonary drug delivery, olfactory displays, and micro or nanoparticle synthesis [1,2,3,4,5,6,7,8,9,10,11]. 

However, SAW devices based on LiNbO_3_ without heat dissipation, even just a few watts, often break down. Fu et al. (2012) studied the ZnO SAW atomization device with an operating frequency of 23.1 MHz at an input radio frequency (RF) power of 21.2 W and an operating frequency of 37.2 MHz and found that a much higher power (>20 W) was required for atomization [12]. Guo et al. (2015) investigated the ZnO SAW atomization device, which was excited with frequencies ranging from 37.2 MHz to 63.3 MHz, and also found that a much higher power (>20 W) was required for atomization [13]. With such a large input power, it was difficult to miniaturize the RF source to be hand-held. More importantly, the aerosol droplets generated during the atomization condensed in the air and dripped on the inter-digitated transducer (IDT), causing the electrodes to short-circuit [14,15,16,17,18,19,20,21,22,23]. This is one reason prevented the commercial application of SAW atomization.

To overcome the electrode-waterproofing problem, this paper proposes an SU-8-2002 film coating on the IDT and investigates the effect of the coating on atomization. SU-8-2002 is a high contrast, epoxy-based photoresistor designed for micromachining and other microelectronic applications. SU-8 2002 is an improved formulation of SU-8, which has been widely used by Micro-electromechanical Systems (MEMS) producers for many years. Film thicknesses of 2−200 microns can be achieved with a single coat process. Moreover, to the best of the authors’ knowledge, there have been no studies thus far regarding the link between LiNbO_3_-based SAW atomization and its heating effects. In order to address the cracking problem of the LiNbO_3_ substrate caused by heat, we used an aluminum heat sink (28 mm × 19 mm × 6 mm) fitted beneath the substrate to enhance heat dissipation. Therefore, the characteristics of SAW atomization behavior based on the SU-8-2002 film, alongside waterproofing-test behavior and the thermal effects, were studied.

## 2. Experiment Approach

### 2.1. SAW Device Design

**Figure 1:**
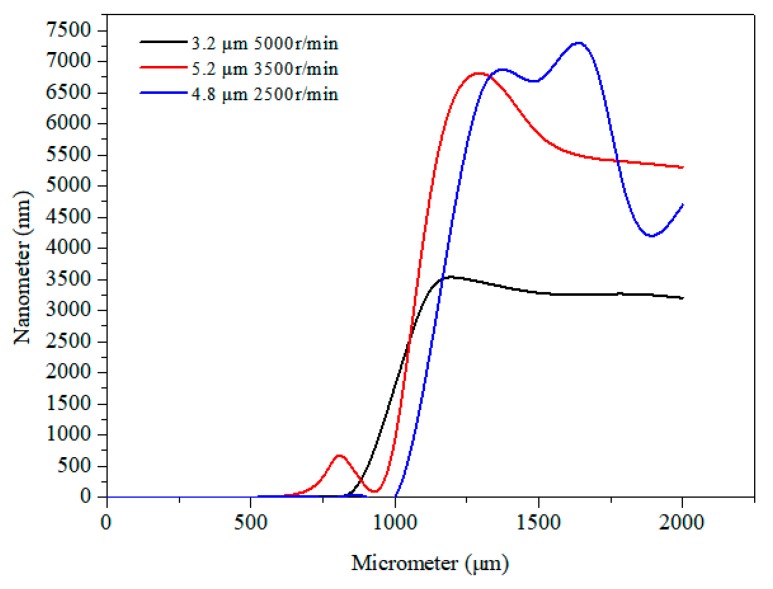
Thicknesses of the film coatings at different spin speeds.

**Figure 2:**
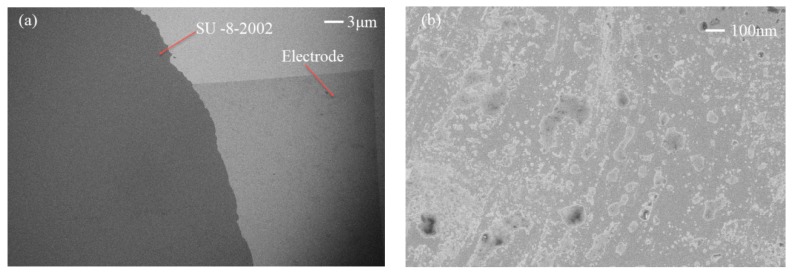
SEM images of the SU-8-2002 film coating. (**a**) The electrode surface at 30× magnification, (**b**) The electrode surface at 1000× magnification.

A SAW device was realized on a 128° Y–X LiNbO_3_ substrate. A 150 nm-thick aluminum electrode consisting of 30 pairs of fingers was fabricated on a substrate using the standard lift-off technique and photolithography techniques. After using acetone, methanol, and deionized water to clean the wafer, the substrate was patterned with a positive photoresistor (AR-P 5350) using a photolithography technique used for making IDT electrode patterns. The substrate was then cleaned with a mild oxygen plasma (Technics). Subsequently, 150 nm of aluminum was sputtered using a RF-magnetron system on the patterned substrate. Finally, the photoresistor stripping was used to achieve the electrode. This design had a wavelength of 130 μm, an aperture of 10 mm, and a center frequency of 30 MHz. To avoid short-circuiting, the thickness of SU-8-2002 needed to be thicker than the IDTs. Limited by the spin speed of the spin-coating machine (LAURELL, WS-650MZ-23NPPB) and the material viscosity of SU-8-2002, the spin speeds were set to 5000 r/min, 3500 r/min, and 2500 r/min, with a normalized thicknesses of SU-8-2002 of 3.2 μm, 4.8 μm, and 5.2 μm, respectively. LiNbO_3_ wafers were then put on a 65 °C hotplate with good thermal control and uniformity to soft-bake for 5 min, followed by the wafers being put on a 95 °C hotplate to bake for 15 min, after which the wafers were removed from the hotplate and allowed to cool to room temperature. Figure 1 shows the results of the film-coating test using the Probe profilometer (VEECO, Dektak 150). However, increasing the spin speed to a maximum of 8000 r/min did not decrease the film thickness further. Figure 2 shows three typical SEM images of the electrode surface with the film coating. Figure 2a shows an image of the electrode surface coated with SU-8-2002 at 30× magnification, suggesting the surface was well-coated with the film. Figure 2b shows the electrode surface at 1000× magnification, suggesting that a small number of nanoparticles accumulated on the surface.

### 2.2. Experimental Setup

**Figure 3:**
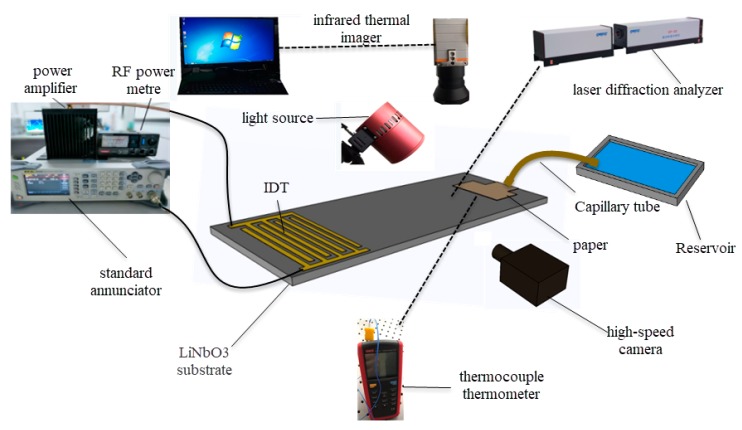
Experiment platform.

In order to evaluate the dispensing performance of the film coating, an observation system and a measurement system were employed, as shown in Figure 3. RF signals from a standard annunciator (RIGOL DSG3000) were amplified by a power amplifier (Mini-circuit LZY-22) and fed to the IDT. A RF power meter was used to measure the amplified RF signal power before connecting the IDT, and a polyester-cellulose paper (C1, Lymtech Scientific, USA) was used to draw liquid from a reservoir for continuous atomization. A high-speed camera (Optronis CP70-1HS-M-1900) with a frame rate of up to 10,000 frames per second was connected to a fixed-focus lens with a magnification of 0.7–4.5 times (Photron D00545) and used to clearly observe the whole atomization phenomenon. A laser diffraction analyzer (OMCC, DP-02) was used to accurately measure the size distribution of the atomized droplets, and the real-time temperature changes of the droplets on the paper were measured using an infrared thermal imager (Gobi-640-GigE) and a thermocouple thermometer (UT325). The resonance frequencies of the SAW device were measured using a network analyzer (E5073C, Agilent).

## 3. Results and Discussion

### 3.1. Waterproof Test and the Influence of the Film Coating on Energy Radiation from the SAW 

**Figure 4:**
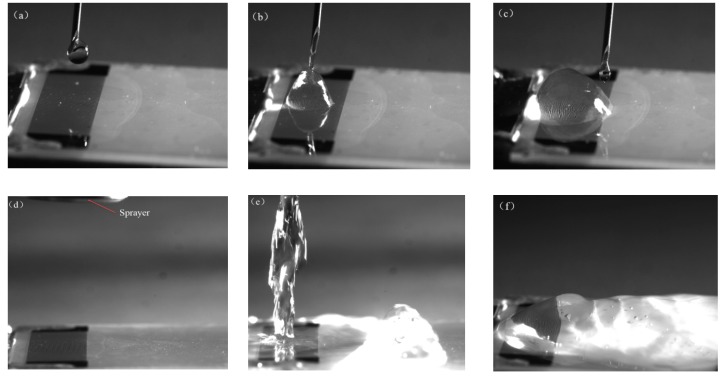
Waterproof performance tests of the surface acoustic wave (SAW) device with the film coating. (**a**–**c**) The electrode dripping test. (**d**–**f**) Corresponding images of the electrode water-spraying test.

In this section, the waterproof performance tests of the SAW devices with the film coating were investigated. Figure 4a–c shows the dripping test on the electrode where the film-coating thickness was 3.2 μm. A syringe dripped the droplet vertically onto the IDT electrode, while the IDT electrode communicated with the signal. This process lasted 10 min, and the drip rate was 5 mL/min. In this process, the liquid vibrated on the electrode and then constantly moved smoothly across the surface. If the electrode was not protected, the droplet would immediately cause the electrodes to short-circuit and crack. When the experiment was repeated at different dripping angles, the surface of the electrode was shown to be robust. Figure 4d–f shows how the water was sprayed onto the electrode, covering the surface of the chip with a large amount of liquid. After applying the SAW power, a large amount of liquid vibrated on the surface. During the low power experiment, the SAW energy dissipated in a large amount of liquid and did not appear to move the liquid. This process lasted 1 hand the film coating was not damaged. Similar phenomena appeared in other coating experiments with different film thicknesses.

**Figure 5:**
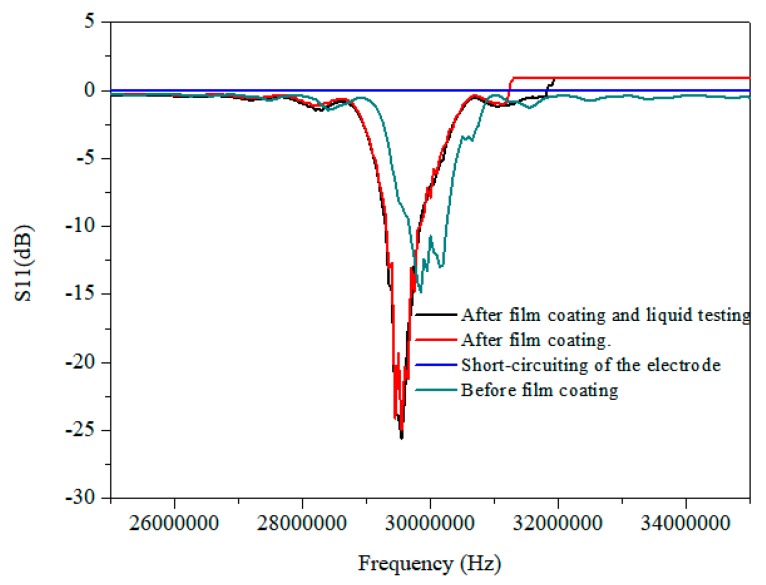
The frequency domain characteristic results under different conditions.

Figure 5 shows the frequency domain characteristic results under different conditions. Before film coating, the center frequency of the fabricated device was 29.80 MHz due to a manufacturing process error and the reflection spectrum (S11) was −15 dB. The film coating of SU-8-2002 changed the impedance of the device; therefore, the center frequency changed from 29.80 MHz to 29.55MHz, as illustrated in Figure 5, which represents a film coating of 3.2 μm. It was observed that in the frequency domain characteristic results of the short-circuiting of the electrode, which was caused by dripping the droplet onto the unprotected electrode, there were no visible resonant frequencies. Compared with this, after film coating and liquid testing, the electrode worked normally. It should be pointed out that the resonant frequencies were kept constant after liquid testing, which was consistent with the pre-testing, and that the reflection spectrum of film coating decreased by around 10 dB. Thus, the electrode protection appeared to be successful. The waterproof performance was tested several times on the other SAW devices with different coating thicknesses, indicating that the film coating on the electrode was robust.

**Figure 6:**
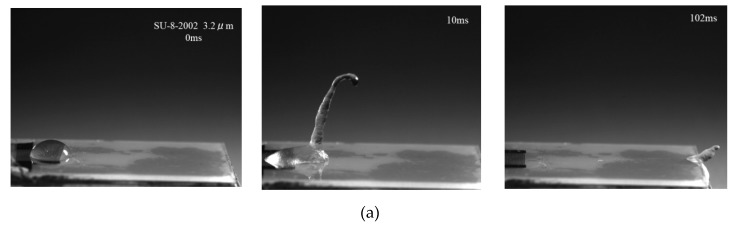

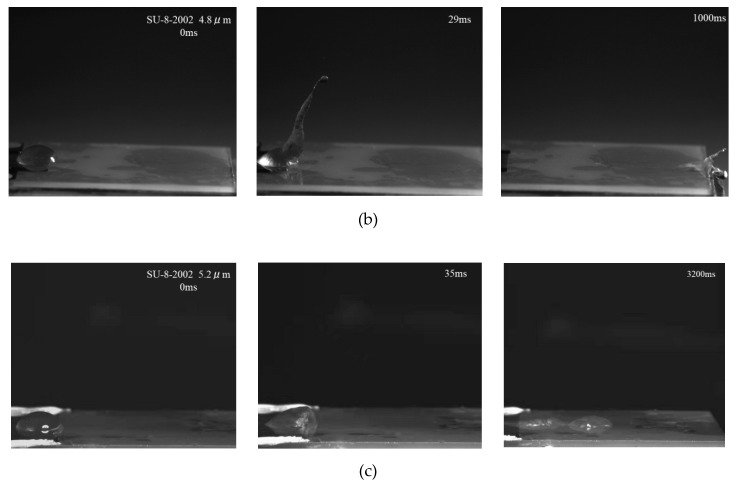
Waterproof performance test of SAW devices with different coating thicknesses. (**a**), (**b**), and (**c**) represent the thicknesses of 3.2 μm, 4.8 μm, and 5.2 μm, respectively.

A 10 μL water droplet was deposited directly onto the IDT via micro-syringe. Figure 6a–c shows the movement of the droplet on the SAW device surface before and after interaction with a SAW at a center frequency of 30 MHz and an RF power of 4.68 W (−7.5 dBm) for different coating thicknesses. Before applying the RF signal, the droplet was at rest on the IDT. After applying the SAW power, the IDT generated a pressure gradient that caused the droplet to deform into a conical shape; as the energy in the droplet increased, the droplet began to creep. As shown in Figure 6a, with a coating thicknesses of 3.2 μm, the undulation of the droplet surface led to the elongation of a crest at 10 ms, then crept to the end of the device, where it began to eject droplets at 102 ms. With increasing coating thicknesses, the time that the droplet took to creep to the end of the device and deform into a conical shape increased, as shown in Figure 6b. With a coating thickness of 5.2 μm, the droplet was observed to vibrate, but without forming a crest at 35 ms followed by a very slow creep on the surface of the device; no deformation or ejection occurred at 3200 ms. This was because as the thickness of the coating increased, the film absorbed the energy of the SAW. The following sections discuss the effect of the coating on atomization characteristics and heat dissipation.

### 3.2. Atomization Behavior

**Figure 7:**
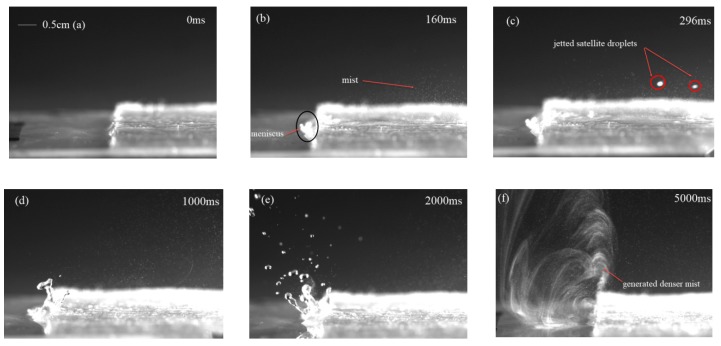
Atomization behavior of 3.2 μm SU-8-2002 interdigitated transducer (IDT) coating thicknesses with a SAW frequency of 30 MHz at a power of 4.86 W. (**a**) 0 ms, (**b**) 160 ms, (**c**) 296 ms, (**d**) 1000 ms, (**e**) 2000 ms, and (**f**) 5000 ms.

**Figure 8:**
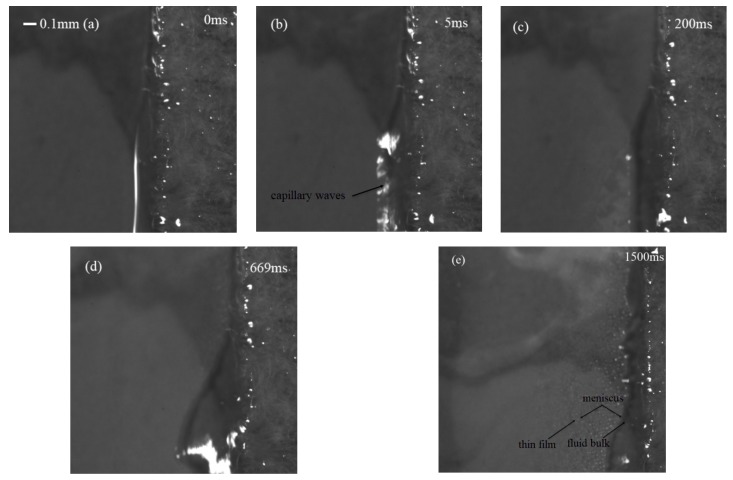
A high-magnification image of the movement of the liquid on the paper. (**a**) 0 ms, (**b**) 5 ms, (**c**) 200 ms, (**d**) 669 ms, (**e**) 1500 ms.

Figure 7 shows the whole process of the water atomization under the action of SAW with an IDT coating thickness of 3.2 μm, where the SAW device was driven by an RF signal power of 4.68 W (−7.5 dBm). The polyester fiber-paper strip was placed on the surface of the device to carry the liquid, and under the action of the SAW, the liquid was pulled out of the paper toward the SAW for atomization. Due to Schlichting streaming, a meniscus film was formed at the front of the strip, as shown in Figure 7b. When the SAW was propagated onto the substrate and encountered the droplet on the propagation path, the SAW energy was coupled onto the droplet and internal streaming was generated. Using paper-based atomization to significantly reduce the characteristic height of the liquid, when the thickness of the liquid was less than the wavelength of the excitation wave in the liquid, the dominant Schlichting streaming dragged the liquid out of the paper, with the thickness of the meniscus gradually increasing and becoming larger than the excitation wavelength. Eckart streaming was dominant and formed capillary waves on the meniscus liquid surface. The capillary waves destabilized the free surface of the liquid and, consequently, the jetting of satellite droplets and a plume of aerosol mist was driven from the surface, as illustrated by Figure 7b,c, in which aerosol particles ranged from tens to hundreds of microns. From the pictures shown in Figure 7d,e, when the volume of liquid increased, a small crest formed at the leading edge of the paper, which then elongated and pinched off, causing a large number of satellite drops to fly away from the free surface. When the liquid was consumed very rapidly, leaving a thin film at the edge of the paper, many smaller aerosol droplets came from the breakup of the free surface and were more violent, as shown in Figure 7f. Figure 8 shows the sequence magnification images of the movement of the liquid at the paper. Initially, the liquid was pulled out of the paper toward the SAW and vibrated, generating a capillary wave on the surface. Schlichting streaming pulled the liquid out of the paper, forming a meniscus comprised of a thin film and a bulk region. The jetting of the satellite droplets came from the bulk region, while the denser mists resulted from the thin film; this was caused by the different characteristic lengths and capillary frequencies.

**Figure 9:**
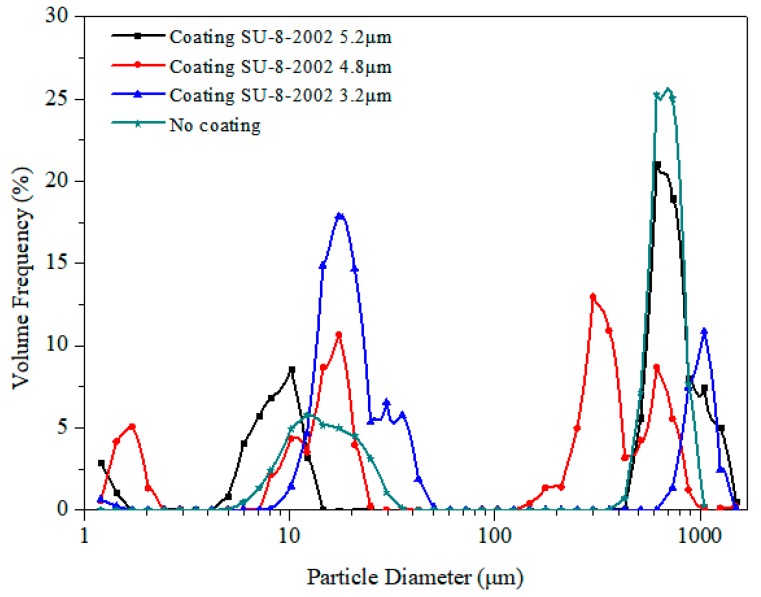
The volume-based droplet size distribution of atomization at 4.68 W with a 30 MHz excitation radio frequency (RF signal), measured with a laser diffraction analyzer.

Aerosol size distribution is an important characteristic of atomization. The mean diameter can be expressed based on the balance between the viscous and capillary force [21]:

(1) $$D∼\lambda ∼\gamma H^{2}/\mu fL^{2}$$

where *D* is the mean diameter, *λ* is the wavelength of the capillary wave, *γ* is the surface tension, *μ* is the viscosity, *H* and *L* represent the height and length of the parent drop or film, and *f* is the capillary frequency. In the experiment, the liquid had a viscosity *μ* of 10^−3^ kg/ms and a surface tension *γ* of 10^−2^ N/m. The bulk region had a capillary frequency *f_c_* of 104 Hz and *H/L* close to 0.1 [9]. When these values are substituted into Equation (1), the prediction of *D* is around 10 μm. When the thickness of this region increases, i.e., *H*/*L* increases from about 0.5 to 1, then the prediction of *D* is above 100 μm. In the thin film, *L* is equal to 2λ_SAW_, *H* ≈ 7/8 λ*_l_* *cosθ_R_, where λ*_l_* represents the sound wavelength, *θ_R_* ≈ 22°, and the capillary frequency *f_c_* is 10^6^ Hz. When these values are substituted into Equation (1), the prediction of *D* is around 1 μm. The particle size distribution was measured with a laser diffraction analyzer. Figure 9 shows the volume-based droplet size distribution. In all cases, the particle size distribution showed trimodal distribution, which was consistent with previous studies (Qi et al., 2008, 2010; Wang and Hu, 2016). The film coating on SU-8-2002 had little effect on the particle size distribution. There were three peaks in this case: The first peak was around 1–2 μm, the second peak was around 10–30 μm, and the third peak was above 100 μm. The largest droplets with diameters above 100 μm were generated by jetting the satellite droplets, which were ejected from the bulk region due to the direct interaction of SAW with the liquid, while the second peak of droplets was governed by Eckart streaming, with the droplets coming from the meniscus. At this stage, the Eckart streaming caused the capillary waves on the meniscus liquid surface; as the energy increased the capillary waves became unstable. The third range of droplets was due to Schlichting streaming, where the diameter of a droplet was determined by the inertia–capillary resonance, thereby producing a smaller mist than the previous stage.

**Figure 10:**
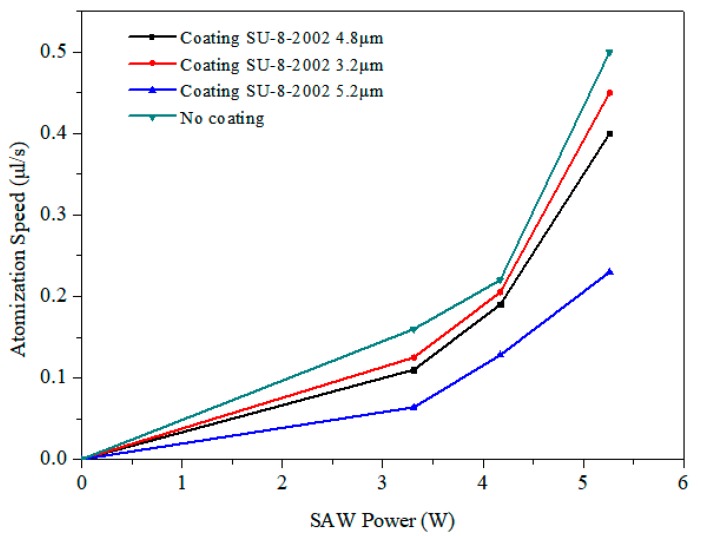
Atomization speed (i.e., droplet volume divided by the total atomization) over the SAW power.

The atomization speed as a function of the SAW power is shown as Figure 10. In general, with the increase in power, the atomization speed increases significantly. In the case of no film coating, the atomization rate at 5.26 W was 0.5 μL/s, while with a film coating of 3.2 μm at the same power, the atomization rate was 0.45 μL/s. However, with a film coating of 5.2 μm, the atomization rate decreased dramatically to 0.23 μL/s.

### 3.3. Heating Effects

Heat was generated from the activity of the SAW device. This caused a breakdown or cracks in the LiNbO_3_ SAW device due to the brittleness of the ceramic material under a large thermal shock, a problem which has continuously plagued researchers.

**Figure 11:**
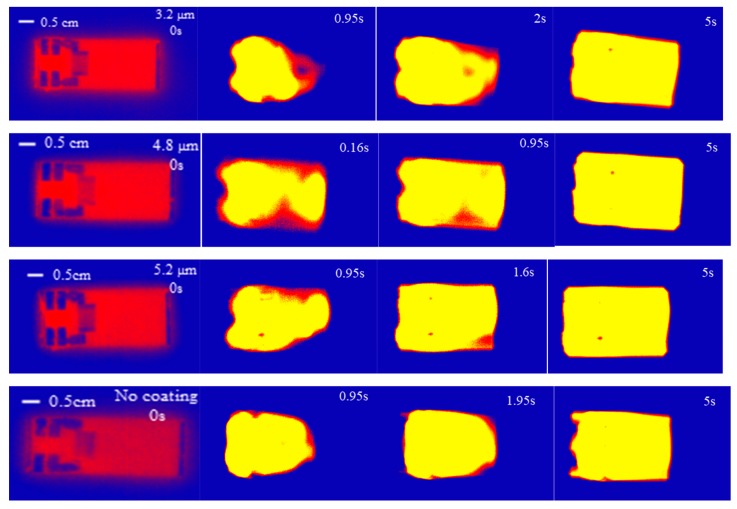
The thermal effect distribution of the device under different coating thicknesses.

**Figure 12:**
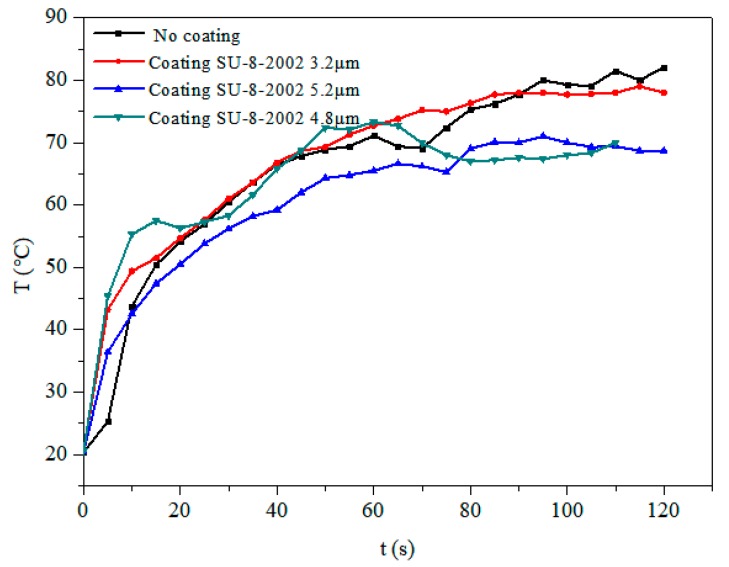
Temperature changes of the atomized liquid under different coating thicknesses.

Researchers found that the main thermal effect caused by temperature changes on a LiNbO_3_ surface without any heat sink was so great that it cracked easily. We therefore placed a 28 mm × 19 mm × 6 mm aluminum heat sink behind the LiNbO_3_ substrate to improve the heat distribution. The SAW device was excited with a power of 4.68 W and a central frequency of 30 MHz, and an infrared thermal image was used to study the thermal effect distribution of the device under different coating thicknesses. It can be seen from Figure 11 that all of the devices reached uniform temperatures within 5 s, although, at the beginning, the temperature of the film coating part was higher than the other parts of the surface. In order to study the temperature changes of the liquid based on the strip atomization, a thermocouple thermometer was used to measure the temperature of the atomized liquid at the front edge of the paper. The temperature of the atomized liquid under different coating thicknesses is shown in Figure 12. It was observed that the temperature initially increased rapidly and gradually reached a steady state. With no film coating, the temperature increased to 82 °C. As the thickness of the film coating increased, the temperature decreased a little in comparison to no coating; the temperature of the 3.2 μm film coating increased to 78 °C within 2 min. Under these conditions, the heating effect did not contribute much to the atomization process. Thus, using a heat sink was shown to allow the lithium niobate substrate to reach a uniform temperature immediately while preventing the surface from cracking. Moreover, the film coating of SU-8-2002 had little effect on the temperature uniformity.

## 4. Conclusions

In summary, the problem of aerosol droplets generated during atomization condensing in the air and dripping on to the IDT, thereby causing the electrodes to short-circuit, was solved using coating with a SU-8-2002 film. This study investigated the effect of the SU-8-2002 film on atomization behavior and heating through an experimental approach. During the atomization process, a meniscus film formed at the front of the strip and a capillary wave formed on the meniscus liquid surface, thereby destabilizing the free surface of liquid and, consequently, the jetting of satellite droplets. The particle size distribution showed trimodal distribution, which the film coating of SU-8-2002 had little effect on. When the film coating had a thickness of 3.2 μm, the atomization rate was 0.45 μL/s, whereas, when there was no film coating, the atomization rate was 0.5 μL/s at a power of 5.26 W. Investigation of the thermal effect revealed that it did not contribute much to the atomization process in the aforementioned set ups. In addition, SAW devices with SU-8-2002 film coatings have the potential for commercial applications and for integration with other microfluidic systems.
